# Automated Classification of Idiopathic Pulmonary Fibrosis in Pathological Images Using Convolutional Neural Network and Generative Adversarial Networks

**DOI:** 10.3390/diagnostics12123195

**Published:** 2022-12-16

**Authors:** Atsushi Teramoto, Tetsuya Tsukamoto, Ayano Michiba, Yuka Kiriyama, Eiko Sakurai, Kazuyoshi Imaizumi, Kuniaki Saito, Hiroshi Fujita

**Affiliations:** 1 School of Medical Sciences, Fujita Health University, Toyoake 470-1192, Japan; 2 Graduate School of Medicine, Fujita Health University, Toyoake 470-1192, Japan; 3 Faculty of Engineering, Gifu University, Gifu 501-1194, Japan

**Keywords:** idiopathic interstitial pneumonias, classification, convolutional neural network, generative adversarial networks

## Abstract

Interstitial pneumonia of uncertain cause is referred to as idiopathic interstitial pneumonia (IIP). Among the various types of IIPs, the prognosis of cases of idiopathic pulmonary fibrosis (IPF) is extremely poor, and accurate differentiation between IPF and non-IPF pneumonia is critical. In this study, we consider deep learning (DL) methods owing to their excellent image classification capabilities. Although DL models require large quantities of training data, collecting a large number of pathological specimens is difficult for rare diseases. In this study, we propose an end-to-end scheme to automatically classify IIPs using a convolutional neural network (CNN) model. To compensate for the lack of data on rare diseases, we introduce a two-step training method to generate pathological images of IIPs using a generative adversarial network (GAN). Tissue specimens from 24 patients with IIPs were scanned using a whole slide scanner, and the resulting images were divided into patch images with a size of 224 × 224 pixels. A progressive growth GAN (PGGAN) model was trained using 23,142 IPF images and 7817 non-IPF images to generate 10,000 images for each of the two categories. The images generated by the PGGAN were used along with real images to train the CNN model. An evaluation of the images generated by the PGGAN showed that cells and their locations were well-expressed. We also obtained the best classification performance with a detection sensitivity of 97.2% and a specificity of 69.4% for IPF using DenseNet. The classification performance was also improved by using PGGAN-generated images. These results indicate that the proposed method may be considered effective for the diagnosis of IPF.

## 1. Introduction

### 1.1. Background

Interstitial pneumonia is an inflammation that occurs in the interstitium between alveoli. There are many types of interstitial pneumonia, including interstitial pneumonia of unknown cause, which is referred to as idiopathic interstitial pneumonia (IIP). IIPs include a variety of pathological forms, with idiopathic pulmonary fibrosis (IPF) having the poorest prognosis [1]. Differentiation of IPF is important because the associated treatment methods differ significantly from those of other IIPs (non-IPF).

In the diagnosis of IIPs, a high-resolution chest CT scan is used to diagnose the extent and type of inflammation. If differentiation is difficult on a CT examination, pathological examination is performed using lung biopsy. Recently, whole specimens have been digitized using whole slide scanners, and digital images have become more common for diagnosis [2]. However, the diagnosis of IPF using pathological imaging requires extensive experience and very few pathologists are capable of performing the task. Therefore, we aimed to develop assistive technology to diagnose IPF accurately in this study.

Artificial intelligence (AI) has evolved markedly with the emergence of deep learning (DL) in the early 2010s and has shown superior capabilities in image recognition [3,4]. Therefore, in this study, we focused on the classification of IPF and non-IPF patients using DL.

### 1.2. Related Works

Various DL technologies have been proposed for lung diseases [5,6,7,8,9,10]. We developed a method to automatically detect lung nodules in CT images using a convolutional neural network (CNN), which is a DL technique [5]. For pathological images, an automated method for classifying histological types of lung cancer cells by CNN and a method for differentiating between benign and malignant cells have also been proposed [6,7,8]. Regarding pneumonia, many studies have considered the automatic detection of COVID-19 pneumonia [9] and predicted the severity of the disease [10]. In a study using non-lung histopathology specimens, Shi et al. proposed a method for the automatic detection of gastric cancer regions in images of gastric histopathology specimens using a CNN decoder for feature extraction and an attention mechanism [11]. The evaluation using two datasets showed that it has a satisfactory detection agreement. Li et al. also proposed a classification method for cholangiocarcinoma by introducing a generator and a discriminator with a transformer [12].

To detect and classify IIPs, Takeuchi et al. studied the automatic extraction of regions of IIPs from CT images and their classification as IPF or non-IPF, and achieved a differentiation accuracy of 75.7% [13].

Uegami et al. used pathological images to automatically classify IIPs by extracting features from finely cropped images using self-supervised learning [14]. The features were clustered, and pathologists manually merged clusters with the same pathological features. On this basis, they developed a method to classify IIPs using deep learning, which achieved an AUC of 0.92. Their study demonstrated the feasibility of analyzing pathological patterns of IIPs using deep learning. In contrast, the model’s ability to automatically classify IIPs without manual clustering by pathologists exhibited an AUC of 0.65. To the best of our knowledge, no other works in the relevant literature have considered this approach, and no existing methods can automatically classify IIPs with sufficient accuracy. 

Thus, very few studies have classified pathological images of IIPs, and automated classification remains challenging. IIP is a rare disease, and so collecting a large number of biopsy specimens is difficult. Despite the small number of cases, the patterns that emerge in tissue specimens from IIPs are diverse. Therefore, methods that can produce satisfactory results using data on a small number of cases are needed.

In this study, we consider data augmentation via a generative adversarial network (GAN) model, which can generate a large number of images that resemble patterns learned during an adversarial training process. These methods attracted considerable attention as a groundbreaking approach when they were originally developed. In prior works, we used GAN models to classify lesions. For example, we proposed a conditional info-GAN designed to generate CT images of lung nodules with various shapes, and showed that the generated images can be used to improve the accuracy of lung cancer histology classification [15]. In addition, we automatically generated lung cell images using progressive growth GANs, which can generate high-resolution images, and applied the generated outputs to classify benign and malignant lung cells. This method achieved results better than those obtained without using the generated images [16]. In addition, a method to generate MR images of the head similar to those of stroke patients from MR images of healthy patients using a CycleGAN model was proposed. This approach was then used to improve the accuracy of automated detection of cerebral infarction using the transformed images [17].

### 1.3. Objective and Contributions

Based on the background and challenges described above, we propose a method to automatically classify pathological specimens of IIPs. The main contributions of this study are as follows.

1. Image generation using GANs is introduced for use as input data for classification. By using GAN with progressive growing mechanism, high-resolution images are generated in a stable manner. Generated images can improve the classification accuracy of IIPs.
2. The CNN model used for classification is trained in two steps: a rough pretraining using generated pathological images, and fine tuning using real images to obtain high accuracy.

## 2. Method

### 2.1. Outline

**Figure 1:**
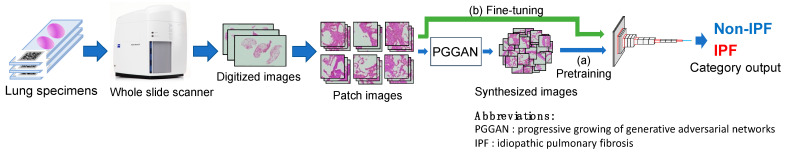
Outline of the proposed method.

An outline of this study is presented in Figure 1. First, tissue specimens from IPF and non-IPF patients were scanned with a whole slide scanner and classified into patch images. Only valid patch images were registered in the image dataset. Subsequently, a GAN was introduced to generate synthesized IPF/non-IPF images and augment the image dataset, and a CNN trained on the GAN-generated images and real images was used to derive the IPF/non-IPF classification results.

### 2.2. Image Dataset

Patients diagnosed with interstitial pneumonia who were biopsied at the Fujita Medical University Hospital were included in this study. Of these patients, 12 were confirmed to have IPF, and 12 were diagnosed as non-IPF. The final diagnosis for patients without IPF consisted of non-specific interstitial pneumonia (NSIP), cryptogenic organizing pneumonia (COP), and pulmonary involvement associated with collagen vascular disease. Specimens were obtained by thoracoscopic lung biopsy and prepared by HE staining. The dataset included 23 patients with IPF and 33 non-IPF patients. 

Images of the specimens were collected using a whole slide scanner (AxioScan Z1, Carl Zeiss, Oberkochen, Germany), tiled with a microscope camera mounted with a 20× objective lens at a pixel resolution of 0.22 μm. The images were stored in CZI format with a 24-bit depth and compressed using the JPEG XR image compression algorithm. The areas of tissue present inside the specimen were scanned, resulting in a matrix count of 72, 200 × 59, 600–229, 200 × 102, 700 for the stored images.

### 2.3. Image Preprocessing

**Figure 2:**
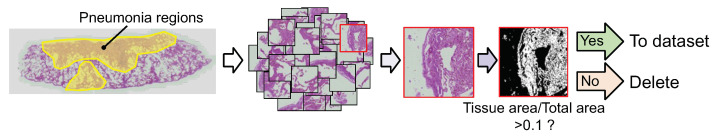
Preparation of patch images.

As the whole-slide image of the pathology specimen was large, it was divided into smaller patch images for processing by deep learning, and the image dataset was constructed using a two-step selection process (Figure 2).

First, because normal lung tissue is likely to impede the classification of IPF and non-IPF, the area of pneumonia in the image was designated by the pathologist, and the region of interest was defined within that area. The whole slide images were then divided into patch images with a size of 2240 × 2240 pixels and raster scanning to avoid overlapping. These patch images also contain many background areas with no tissue. To remove patch images with no tissue, patch images were binarized using Otsu’s binarization algorithm such that the background was black and areas with tissue were white. Images with less than 10% of the total area showing tissue were excluded from processing. These processes resulted in 23,142 IPF and 7817 non-IPF images being registered in the image dataset for this study.

**Figure 3:**
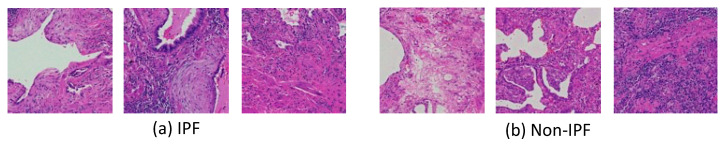
Example images of typical IPF (**a**) and non-IPF (**b**) lesions.

Examples of typical IPF and non-IPF patch images are shown in Figure 3. Typical IPF lesions show a fibrotic pattern. However, there are many atypical ones, and it is often difficult to distinguish them from non-IPF patterns caused by a variety of factors.

### 2.4. Data Augmentation by Generative Adversarial Networks

In this study, we employed pathology specimens from 24 cases of rare diseases, which is a small number for deep learning models. Although the total number of images exceeds 30,000, there was little variation in the images, and there was some concern that overfitting could degrade the classification performance. Therefore, we introduced data augmentation to increase the amount of training data by artificially generating images similar to each class. Simple data augmentation methods generate new images using simple manipulations such as image rotation or scaling. This contributes slightly to an increase in processing performance; however, the performance improvement that can be achieved with these methods is limited because they do not significantly change the image pattern itself.

Recently, the use of GAN models, an image synthesis technique, to generate images similar to real images for deep learning has been widely investigated [18]. Onishi et al. demonstrated that the use of artificial nodule images generated by a GAN model in addition to actual CT images for automated differentiation of benign and malignant nodules on chest CT images improved the performance of nodule differentiation. In this study, we also aimed to improve classification performance by using GAN-generated images.

The progressive growth of GANs (PGGAN) [19], which can generate high-resolution images, has been used as the GAN for data augmentation. Teramoto et al. used a PGGAN model to automatically generate high-resolution lung cell images and used them in the classification process to improve the performance of differentiating between benign and malignant lung cytology images. The results showed that the classification accuracy improved [16]. 

**Figure 4:**
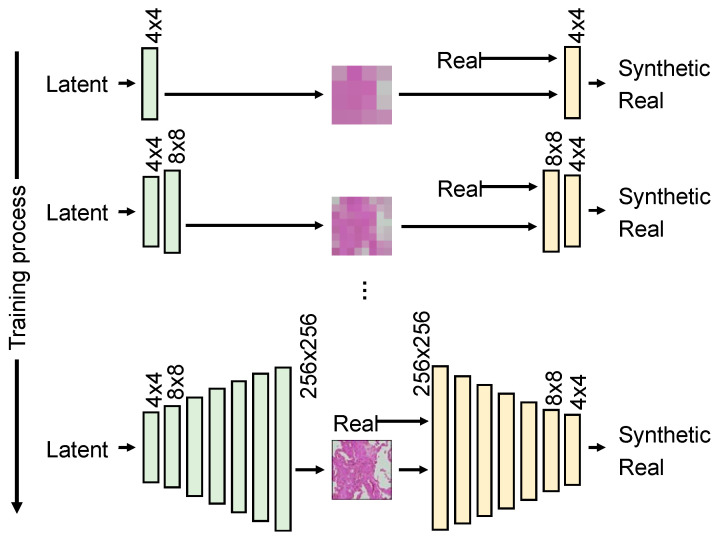
Architecture of PGGAN.

As shown in Figure 4, PGGAN gradually increases the resolution of a GAN trained to produce low-resolution images. Initially, a 4 × 4 pixel image was generated in two convolution layers with 128 random values (latent vectors). The discriminator identifies the real and generated images using two convolution layers and one fully connected layer. Then, by adding two convolution layers to the network of generators and discriminators, the system was adapted to generate 8 × 8 and 16 × 16 pixel images. This process was repeated seven times to produce a 256 × 256 pixel image; the base structure of PGGAN was based on WGAN-GP [20] and was trained using the Wasserstein distance with added gradient penalty. Adam was used as the optimization algorithm, the learning coefficient *lr* was set to 0.00001, *β*_1_ to 0.9, and *β*_2_ to 0.999, and training was performed for 50 epochs.

Using these methods, two PGGANs were created to generate IPF and non-IPF images. The two PGGANs were then assigned random values, and 10,000 images were generated for each. These were subsequently used to train the CNN.

To evaluate the image quality of the images generated by the PGGAN model, we also generated images using a conventional deep convolutional GAN (DCNN) [21] without a progressive mechanism. The DCGAN comprised a generator with five convolutional layers and four scaling layers, as well as a discriminator with six convolution layers and a single fully connected layer to generate images with a size of 256 × 256 pixels from 128 latent vectors. The model was trained over 2000 epochs using the Adam optimizer with a learning coefficient *lr* set to 0.00002, *β*_1_ to 0.9, and *β*_2_ to 0.999, and training was performed for 2000 epochs. 

### 2.5. Two-Step Image Classification

Pathological images were classified as IPF and non-IPF using a CNN model. To increase the variation in images, we prepared images generated by the PGGAN in addition to real images, which were used for training in two stages as described below [16,18]. Note that the image matrix size was resized to 224 × 224 pixels when the images were provided to the CNN.

**Figure 5:**
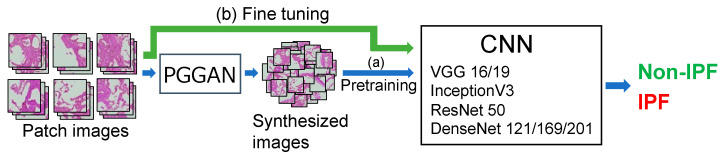
Two-step training of CNN.

First, the CNN for classification was trained using images generated by the PGGAN ((a) in the Figure 5). However, they reproduced the shape, color, and arrangement of cells in a real image, and we considered that studying pathological images using PGGAN-generated images to understand their general characteristics could be useful. Therefore, the CNNs were pretrained using PGGAN-generated images. Several CNN models (VGG-16/19 [22], InceptionV3 [23], ResNet-50 [24], and DenseNet-121/169/201 [25]) were pretrained using the ImageNet database, and the fully connected layer was replaced by a multilayer perceptron with 1024 and 2 units.

These models were trained with 10,000 generated patch images for each IPF and non-IPF case. The Adam optimization algorithm was used, with a learning coefficient *lr* of 0.00001, *β*_1_ of 0.9, and *β*_2_ of 0.999, and training was performed for 50 epochs. These parameters were set so that the training loss was sufficiently reduced, and the validation loss did not increase in the preliminary experiments.

Next, fine-tuning was performed on the CNN pre-trained using the generated images and real images ((b) in the Figure 5). As in a previous study [7], data augmentation was performed by rotating and flipping the images during the training. Because the number of real images was unbalanced between the two classes, the rotation angle was adjusted according to the class to achieve a balance. Consequently, 23,142 IPF cases and 23,451 non-IPF cases were used for the training.

In addition, as an ablation study, a CNN model trained on the ImageNet database without data augmentation by PGGAN was trained by transfer training on actual pathological images and compared with the proposed method.

### 2.6. Visualization

In this study, pathological specimens were divided into patch images and CNN-based classification was performed on a patch image basis. However, there were cells with various morphologies in these specimens. Therefore, to classify using the information of the entire specimen, the probability of IPF when the patch images were identified by the CNN was converted into color information, and the color map output was superimposed on the specimen. Pathologists can use the color map and pathology image for diagnosis, while simultaneously observing them.

### 2.7. Evaluation Metrics

The classification performance of the proposed method was evaluated using image- and case-based methods. In the image-based evaluation, performance was evaluated by tabulating the probability of IPF obtained by providing patch images to the CNN. For case-based classification, we calculated the average value of the probabilities of CNN-output IPF in each case. Based on this value, IPF and non-IPF were classified.

A cross-validation method was used to evaluate the classification ability [26]. In the cross-validation, the image dataset was divided into K subsets to avoid case fragmentation. The CNN was then trained on K-1 subsets; the image data belonging to the remaining subset were defined as test data, and the classification results were evaluated. In the cross-validation method, the test results for all data were obtained by training and testing K times, while changing the subset used as the test data. The data were divided into five subsets (5-fold cross validation) and the classification ability was evaluated.

A confusion matrix was created based on the classification results of all images obtained by cross-validation. Based on the matrix, IPF cases were assumed to be positive and non-IPF cases were assumed to be negative, and the sensitivity, specificity, and accuracy were calculated. We also generated receiver operating characteristic (ROC) curves by determining the true positive fraction (TPF), which is the proportion of correctly diagnosed IPF, and the false positive fraction (FPF), which is the proportion of incorrectly diagnosed non-IPF as IPF, while changing the cut-off value for the image-based or case-based IPF probability.

The calculation was performed using the original software written in the Python programming language with an AMD Ryzen9 3950X processor and 128 GB of DDR4 memory. The training and validation of the CNNs were accelerated by using an NVIDIA Quadro RTX 8000 GPU.

## 3. Results

### 3.1. Synthesized Patch Images Using PGGAN

**Figure 6:**
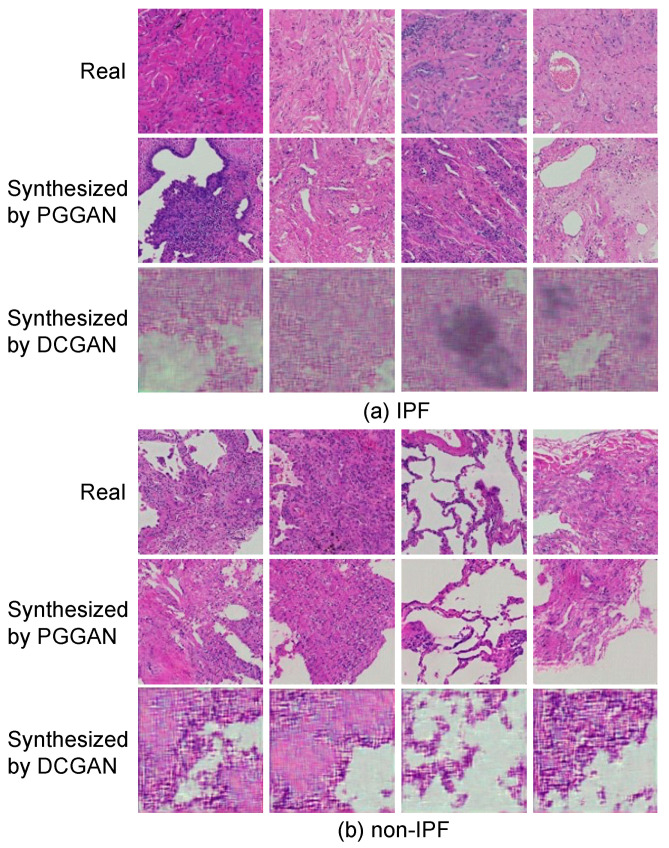
Real and synthesized images generated using PGGAN and conventional DCGAN models. (**a**) Images of IPF; (**b**) Images of non-IPF.

Figure 6 shows a sample synthesis of IPF and non-IPF pathological images using PGGAN and DCGAN, along with real images.

### 3.2. Image Classification Results

**Figure 7:**
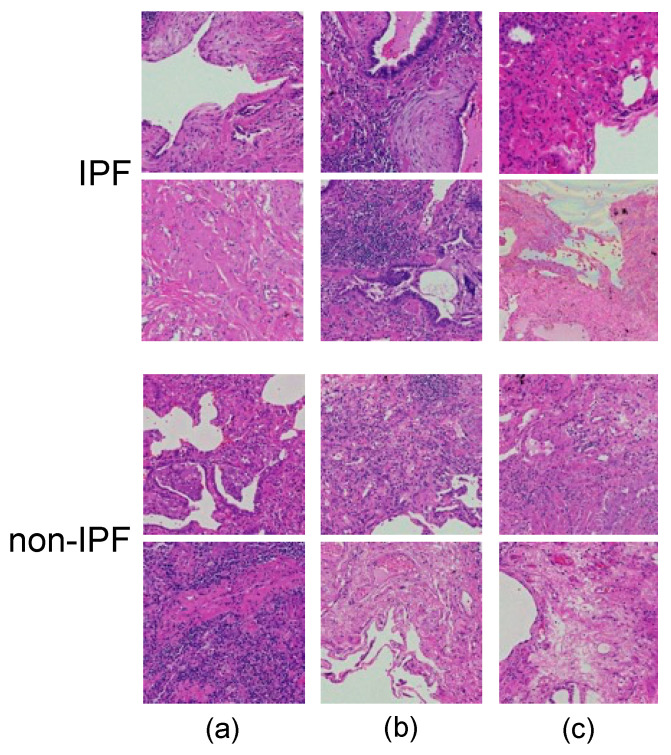
Classified images using the proposed method. (**a**) Correctly classified with and without PGGAN; (**b**) Correctly classified with PGGAN; (**c**) Incorrectly classified with and without PGGAN.

**Figure 8:**
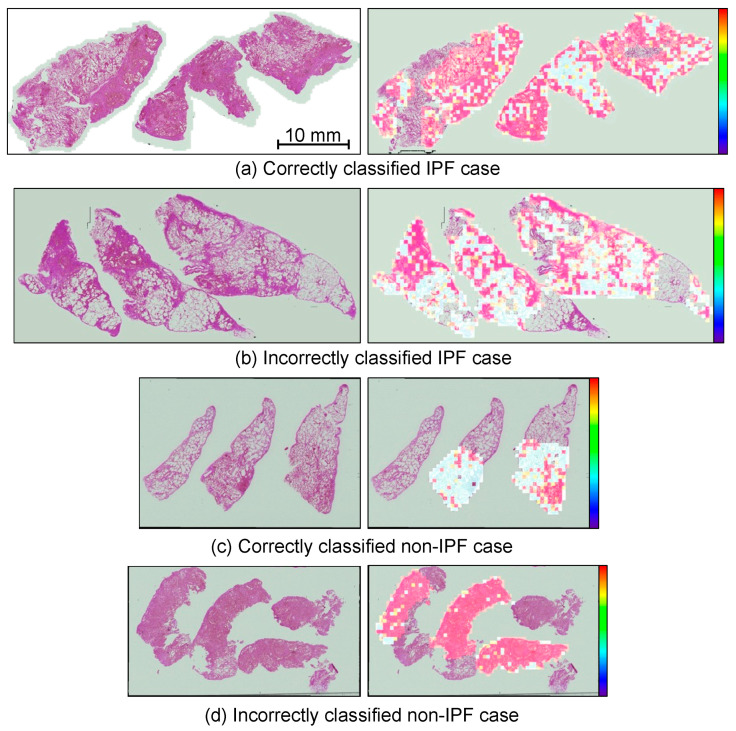
Probability map of classification. Red areas indicate a high probability of IPF. (**a**) Correctly classified specimens of IPF cases; (**b**) Incorrectly classified IPF case; (**c**) Correctly classified non-IPF case; (**d**) Incorrectly classified non-IPF case.

**Figure 9:**
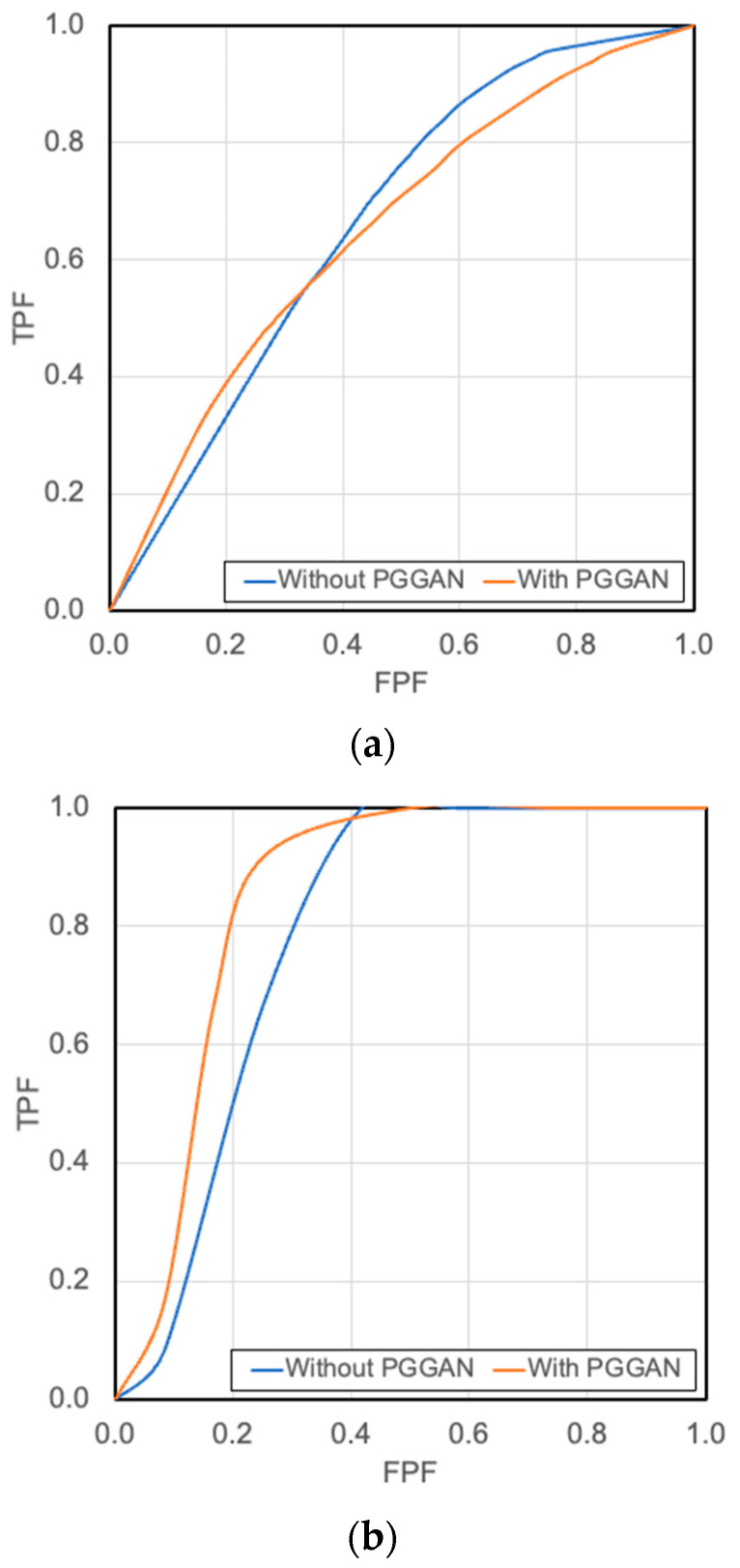
Receiver operating characteristic (ROC) curves: (**a**) Image-based classification; (**b**) Case-based classification.

Figure 7 shows the results of image-based classification with and without data augmentation using the PGGAN model. Next, the probability of a sample showing IPF was calculated for each image, and the results are shown in Figure 8. Table 1 shows the results of the image-based and case-based evaluations of the classification results with the average and standard deviation, and Table 2 shows the confusion matrix of the model with the highest correct rate with and without data augmentation by PGGAN. Figure 9 shows the results of the ROC curves calculated using the two models.

## 4. Discussion

In this study, we developed a method to classify IPF and non-IPF cases by using whole-slide pathology specimen images. Comparing the images generated by the PGGAN and conventional DCGAN models with the real image shown in Figure 6, it may be observed that the DCGAN reproduced an overall color distribution similar to that of the real image, but did not reproduce details such as cells. In contrast, PGGAN accurately reproduced the cells and their arrangement in the histopathological specimen in both IPF and non-IPF cases, and the fibrosis pattern that appears in IPF cases was also observed in the generated images. According to Figure 7, which shows the results of the classification, IPF cases tended to be correctly classified as IPF with a slightly paler color and fibrotic areas. In addition, because several disease classifications were included for non-IPF cases, images without IPF characteristics were classified as non-IPF images. A review of images erroneously classified into different classes showed that, for IPF cases, images with very dark or weak fibrosis were misclassified, whereas for misclassified non-IPF cases, images with IPF-like fibrosis or pale images were included. For misclassified non-IPF cases, images with dark or weak fibrosis were misclassified.

An IPF detection sensitivity of 0.658, specificity of 0.554, and accuracy of 0.632 were obtained using DenseNet-169 without PGGAN data augmentation in image-based evaluation. When using PGGAN data augmentation, a detection sensitivity of 0.691, a specificity of 0.522, and an accuracy of 0.649 were obtained using DenseNet-169. There was no difference in performance between the two groups, as shown by the ROC curve in Figure 9a.

In contrast, the case-based evaluation showed the highest performance when DenseNet-169 was used, with a sensitivity of 0.972, a specificity of 0.583, and an accuracy rate of 0.778 when PGGAN was not used. However, when PGGAN data augmentation was used, the sensitivity was 0.972, the specificity was 0.694, and the accuracy rate was 0.833 with DenseNet-121, indicating that data augmentation with PGGAN contributed to the improvement in classification accuracy. AUC was used as an evaluation measure for comparison with previous studies, and the results showed AUC values of 0.92 for the existing methods that incorporated semi-automated methods, and an AUC of 0.65 for the automated classification. Our fully automated method exhibited an AUC of 0.843, which was higher than that of the previous automated method.

In this work, we introduced multiple CNN models and compared their classification performance with and without data augmentation using a PGGAN. From these results, we found that some CNN models were highly effective with data augmentation, whereas others that were less effective. We assumed that the features to be extracted from the images generated by the PGGAN model differed depending on the structure of the CNN model. Even with only a single CNN, the best architecture generally varied depending on the target image. A comparative evaluation of CNN models is also necessary when using this method in conjunction with PGGAN. As a result of the evaluation, DenseNet exhibited the best performance in combination with augmented data produced by the PGGAN model.

Not all pathologists are able to correctly diagnose IPF, and a cohort study on the diagnostic accuracy of IPF showed that the accuracy of diagnosing IPF patients using pathology and CT images depends on physician experience, and the correct diagnosis rate of IPF is about 0.7 on the C-index [27]. The proposed method has the potential to assist non-specialists in diagnosing IPF and can be used as a tool for resident education and diagnostic assistance because the distribution of lesions can be visualized using probability maps.

This work involves some limitations. For example, we only used specimens collected from a single institution. Further research should collect specimens from many institutions to improve the performance of classification models. In terms of technology, we introduced a PGGAN to generate high-resolution images and compared its performance with that of a DCGAN. Because image-generation technology is rapidly evolving, new image-generation models should be developed and comparative evaluations conducted. Although the training parameters of the CNN used in this study were fixed based on the results of preliminary experiments, automatic adjustment should be considered to obtain higher generalization performance.

In the future, we plan to develop a diagnostic support system that implements the proposed method in an application to clinical practice. For this purpose, whether the proposed approach can contribute to diagnosis by pathologists may be considered, including the reliability of the results. From a technical perspective, in developing diagnostic support applications, it is necessary to evaluate and optimize the computational cost of each process.

## 5. Conclusions

In this study, we have proposed an automated IPF detection method using a convolutional neural network in combination with image generation technology to support the diagnosis of IPF using histopathological specimens. The results of an experimental evaluation have shown that the pathological images generated by the PGGAN exhibited the same characteristics as the real images. When they were used together to classify IIPs using a CNN model, the proposed approach outperformed the conventional method with accuracy and AUC values of 0.833. These results indicate that the proposed method may prove useful in classifying IIPs.

## Figures and Tables

**Table 1:** Performance evaluation.

**(a) Image-Based Classification**					
**CNN Model**	**Data Augmentation**	**Sensitivity**	**Specificity**	**Accuracy**	**AUC**
VGG-16	w/o GAN DA	0.601 ± 0.062	0.547 ± 0.031	0.588 ± 0.050	0.615 ± 0.039
	w GAN DA	0.582 ± 0.040	0.587 ± 0.047	0.583 ± 0.019	0.618 ± 0.009
VGG-19	w/o GAN DA	0.609 ± 0.027	0.592 ± 0.015	0.605 ± 0.021	0.641 ± 0.021
	w GAN DA	0.607 ± 0.006	0.563 ± 0.014	0.596 ± 0.005	0.618 ± 0.007
InceptionV3	w/o GAN DA	0.625 ± 0.020	0.556 ± 0.023	0.608 ± 0.019	0.618 ± 0.032
	w GAN DA	0.595 ± 0.011	0.527 ± 0.004	0.578 ± 0.008	0.592 ± 0.006
ResNet-50	w/o GAN DA	0.614 ± 0.021	0.578 ± 0.011	0.605 ± 0.014	0.619 ± 0.019
	w GAN DA	0.540 ± 0.089	0.483 ± 0.090	0.526 ± 0.044	0.533 ± 0.009
DenseNet-121	w/o GAN DA	0.628 ± 0.018	0.568 ± 0.019	0.613 ± 0.009	0.628 ± 0.009
	w GAN DA	0.683 ± 0.008	0.521 ± 0.016	0.642 ± 0.002	0.644 ± 0.006
DenseNet-169	w/o GAN DA	0.658 ± 0.019	0.554 ± 0.007	0.632 ± 0.013	0.639 ± 0.022
	w GAN DA	0.691 ± 0.010	0.522 ± 0.015	0.649 ± 0.004	0.649 ± 0.004
DenseNet-201	w/o GAN DA	0.652 ± 0.036	0.557 ± 0.058	0.628 ± 0.013	0.632 ± 0.023
	w GAN DA	0.663 ± 0.013	0.548 ± 0.012	0.634 ± 0.007	0.646 ± 0.006
**(b) Case-Based Classification**					
**CNN Model**	**Data Augmentation**	**Sensitivity**	**Specificity**	**Accuracy**	**AUC**
VGG-16	w/o GAN DA	0.806 ± 0.127	0.583 ± 0.083	0.694 ± 0.087	0.701 ± 0.064
	w GAN DA	0.861 ± 0.048	0.611 ± 0.048	0.736 ± 0.024	0.811 ± 0.039
VGG-19	w/o GAN DA	0.806 ± 0.048	0.639 ± 0.048	0.722 ± 0.024	0.765 ± 0.022
	w GAN DA	0.889 ± 0.048	0.639 ± 0.048	0.764 ± 0.024	0.843 ± 0.019
InceptionV3	w/o GAN DA	0.944 ± 0.048	0.583 ± 0.000	0.764 ± 0.024	0.757 ± 0.030
	w GAN DA	0.806 ± 0.096	0.667 ± 0.000	0.736 ± 0.048	0.744 ± 0.040
ResNet-50	w/o GAN DA	0.889 ± 0.048	0.611 ± 0.048	0.750 ± 0.042	0.722 ± 0.047
	w GAN DA	0.611 ± 0.048	0.444 ± 0.048	0.528 ± 0.024	0.522 ± 0.028
DenseNet-121	w/o GAN DA	0.861 ± 0.127	0.583 ± 0.000	0.722 ± 0.064	0.734 ± 0.033
	w GAN DA	0.972 ± 0.048	0.694 ± 0.048	0.833 ± 0.000	0.843 ± 0.005
DenseNet-169	w/o GAN DA	0.972 ± 0.048	0.583 ± 0.000	0.778 ± 0.024	0.786 ± 0.013
	w GAN DA	0.944 ± 0.048	0.639 ± 0.048	0.792 ± 0.042	0.826 ± 0.045
DenseNet-201	w/o GAN DA	0.833 ± 0.220	0.583 ± 0.000	0.708 ± 0.110	0.726 ± 0.074
	w GAN DA	0.833 ± 0.083	0.667 ± 0.083	0.750 ± 0.083	0.809 ± 0.024

DA: Data augmentation.

**Table 2:** Confusion matrix of classification (case-based classification).

**(a) Classification without PGGAN (DenseNet-169)**			
		**Predicted Class**	
		**Non-IPF**	**IPF**
Actual class	Non-IPF	7	5
	IPF	0	12
**(b) Classification Using PGGAN (DenseNet-121)**			
		**Predicted Class**	
		**Non-IPF**	**IPF**
Actual class	Non-IPF	9	3
	IPF	1	11

## Data Availability

The source code and additional information used to support the findings of this study are available from the corresponding author upon request.
